# What is preventable harm in healthcare? A systematic review of definitions

**DOI:** 10.1186/1472-6963-12-128

**Published:** 2012-05-25

**Authors:** Mohammed Nabhan, Tarig Elraiyah, Daniel R Brown, James Dilling, Annie LeBlanc, Victor M Montori, Timothy Morgenthaler, James Naessens, Larry Prokop, Veronique Roger, Stephen Swensen, Rodney L Thompson, M Hassan Murad

**Affiliations:** 1 Center for the Science of Healthcare Delivery, Mayo Clinic, Rochester, MN, USA; 2 Knowledge and Evaluation Research Unit, Mayo Clinic, Rochester, MN, USA; 3 Division of Anesthesiology and Critical Care, Mayo Clinic, Rochester, MN, USA; 4 Division of Heath Care Policy Research, Mayo Clinic, Rochester, MN, USA; 5 Division of Pulmonary, Critical Care, and Sleep Medicine, Mayo Clinic, Rochester, MN, USA; 6 Mayo Clinic Libraries, Mayo Clinic, Rochester, MN, USA; 7 Department of Radiology, Mayo Clinic, Rochester, MN, USA; 8 Division of Infectious Diseases, Mayo Clinic, Rochester, MN, USA; 9 Division of Preventive Medicine, Mayo Clinic College of Medicine, 200 First Street SW, Rochester, MN, 55905, USA

**Keywords:** Preventable harm, Systematic review, Healthcare delivery, Safety

## Abstract

**Background:**

Mitigating or reducing the risk of harm associated with the delivery of healthcare is a policy priority. While the risk of harm can be reduced in some instances (i.e. preventable), what constitutes preventable harm remains unclear. A standardized and clear definition of preventable harm is the first step towards safer and more efficient healthcare delivery system. We aimed to summarize the definitions of preventable harm and its conceptualization in healthcare.

**Methods:**

We conducted a comprehensive electronic search of relevant databases from January 2001 to June 2011 for publications that reported a definition of preventable harm. Only English language publications were included. Definitions were coded for common concepts and themes. We included any study type, both original studies and reviews. Two reviewers screened the references for eligibility and 28% (127/460) were finally included. Data collected from studies included study type, description of the study population and setting, and data corresponding to the outcome of interest. Three reviewers extracted the data. The level of agreement between the reviewers was calculated.

**Results:**

One hundred and twenty seven studies were eligible. The three most prevalent preventable harms in the included studies were: medication adverse events (33/127 studies, 26%), central line infections (7/127, 6%) and venous thromboembolism (5/127, 4%). Seven themes or definitions for preventable harm were encountered. The top three were: presence of an identifiable modifiable cause (58/132 definitions, 44%), reasonable adaptation to a process will prevent future recurrence (30/132, 23%), adherence to guidelines (22/132, 16%). Data on the validity or operational characteristic (e.g., accuracy, reproducibility) of definitions were limited.

**Conclusions:**

There is limited empirical evidence of the validity and reliability of the available definitions of preventable harm, such that no single one is supported by high quality evidence. The most common definition is “presence of an identifiable, modifiable cause of harm”.

## Background

As part of the Hippocratic Oath, *“Primum non nocere”,* the Latin phrase that means "First, do no harm" is a basis for ethics taught in medical school. Preventing harms associated with the delivery of healthcare is paramount to improve patient safety, a key component of overall quality of care. Although much is likely preventable, some harm may be inevitable. For example, post-operative bleeding may occur and be harmful despite impeccable surgical technique. Much current discussion on harm events focuses on hospital acquired conditions (HAC) including the group historically known as “never events”. The term "Never Event" was first introduced in 2001 by Ken Kizer, MD, former CEO of the National Quality Forum (NQF) [1], in reference to particularly shocking medical errors (such as wrong-site surgery) that should never occur. In the UK, a framework has been developed for *Never Events* by the National Patient Safety Agency including a core list of such events (updated regularly) and implementation tools for practitioners. This highlights the international interest in promoting patient safety and reducing harms associated with healthcare delivery [2]. Over time, the NQF list, which is publically reported in some states (e.g. Minnesota) [3], has been expanded to signify adverse events that are unambiguous (clearly identifiable and measurable), serious (resulting in death or significant disability), and *usually* preventable [4]. So even in the context of the NQF “Never Events”, there is acknowledgment that they are not always preventable.

While a goal of zero harm is desirable, this may not always be feasible. Many practicing clinicians are dismayed by general statements about eliminating *all* harm, but may be more willing to engage in discussions about substantially reducing the risk of or eliminating *preventable* harm [5]. Therefore, developing a better understanding of the nature of preventable harm could lead to unambiguous communication and superior stakeholder buy-in. Further, once preventable harm is clearly defined and standardized throughout the healthcare system, policy makers can set up necessary plans that would deal with this pressing issue in a more efficient and reliable fashion.

The scholarly activities in this field include a systematic review conducted by Ferner and Aronson to evaluate the published approaches to assess preventability in drug related adverse effects [6]. They concluded that the existing evidence is limited and suggested an approach based on analysis of the mechanisms of adverse reactions and their clinical features. However, this systematic review did not consider other types of harm and did not seek providing a definition of preventable harm. More recently, similar findings were reported by Hakkarainen et al. They concluded that new instruments for assessing the preventability of adverse drug events need to be developed, or the existing ones be further studied, for a more accurate and precise measure of preventability [7]. Hayward and Hofer reviewed 111 active-care patient deaths and estimated that almost a quarter of them were at least possibly preventable by optimal care [8]. This study did not consider harm other than death and did not seek to provide a definition of preventable harm. Therefore, the aim of this systematic review is to summarize the definitions of preventable harm used in the last decade in the published peer reviewed literature and to assess its conceptualization in healthcare.

## Methods

### Aim

This systematic review aims at surveying the medical literature for existing and emerging definitions of preventable harm. We focused on the last decade to evaluate contemporary definitions.

### Data sources and searches

We conducted a comprehensive electronic search of relevant databases (Ovid Medline In-Process & Other Non-Indexed Citations, Ovid MEDLINE, and Ovid EMBASE) from January 2001 to June 2011 for publications that reported a definition of preventable harm. The search strategy was designed and conducted by an experienced reference librarian (LJP) with input from the study’s principal investigator (MHM). Controlled vocabulary supplemented with keywords was used to search for the topic preventable patient harm. The detailed strategy is available in Additional file 1. In addition, we reviewed the reference sections of eligible studies and available reviews and requested potentially eligible studies from content experts.

### Study selection

Eligible publications were articles that provided 1) a single or multiple definitions of harms associated with health care delivery and 2) defined the preventability of those harms. We included all kinds of harm: specific harm such as drug related adverse events, as well as more general type of harm. We included articles of any type including primary and review studies, including editorials and commentaries as we were interested in evaluating all the existing definitions of multiple stakeholders and how they view preventable harm (including researchers, policy makers and opinion leaders). Moreover, we anticipated that definitions in interventional studies would have been assumed and not necessarily validated; thus, having similar face value as definitions in opinion piece publications. We excluded publications in non-English language.

### Data extraction and quality assessment

Two of the study’s authors (MN, TE) screened all abstracts and titles. Upon retrieval of candidate studies, two reviewers (MN, MHM) examined the full text publications and determined study eligibility. The level of agreement between the reviewers on the assignment of a definition theme for preventable harm in each of the studies was estimated using Cohen’s kappa statistic – a measure of inter-rater agreement. Agreement was described as a binary outcome in a 2x2 table (yes, agreed vs. no, did not agree) and kappa was estimated as the ratio of (observed agreement – agreement expected by chance) to (1- agreement expected by chance). Disagreements were resolved by discussion and consensus of the review team.

### Data synthesis and analysis

The two reviewers, MN and MHM, collected and synthesized the data. Extracted data included type of study, description of the study population and setting, and data corresponding to the outcome of interest (type and severity of harm, and definition of preventability). Reviewers categorized the definitions of preventability encountered in each article into domains (themes). Reviewers developed the classification terms of the domains using a saturation approach (new definitions are added to the list of categories until no new themes were identified). The severity of harm was extracted as reported/classified in the included studies (i.e., using study authors’ designation of severity as a requirement for inclusion of harm in their study). No classification of the severity of harm was feasible.

We also extracted data on the operational performance of the definitions of preventability reported in the included studies (i.e., feasibility of using the definition and assigning a level of preventability, measures of agreement such as kappa statistic among those who assigned a preventability level, any attempts to validate the definition and whether the definition had a reference or citation). We classified agreement based on kappa reported in the studies as poor, fair to moderate, or substantial [8].

## Results

**Figure 1:**
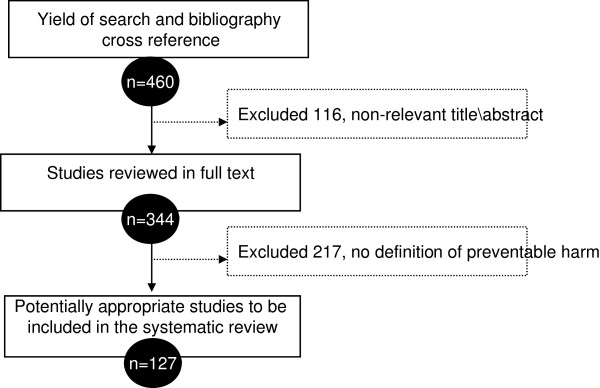
Study selection process.

The literature search yielded 460 references; of which 127 were finally included. Study selection process is depicted in Figure 1. The characteristics and bibliography of included studies are described in Additional file 2: Table S1. Cohen’s Kappa statistic among the systematic review team averaged 0.86. The definitions were described in observational studies (45/129, 35%), expert opinion publications (non-original studies, opinion piece and commentaries) (41/129, 32%), cross-sectional studies (27/129, 21%), systematic reviews (9/129, 7%), consensus statements (4/129, 3%), and case reports, case–control studies and randomized trials (3/129, 2%). Several studies reported the implementation of different interventions aimed at the reduction of patient harm with varying success. These data about interventions were sparse and not analyzed.

### Type of harm

The three most prevalent preventable harms cited in the included studies were medication adverse events 26% (33/127 studies), central line infections 6% (7/127) and hospital-stay related venous thromboembolism 4% (5/127) where medication adverse events are defined as errors in prescribing, delivering or monitoring the effects of the drug, which is different from regular side effects. Most of the studies 76% (96/127) were conducted in inpatient setting. Additional file 3: Table S2 shows the number of articles that used each definition of harm and the type of harm associated with it.

### Definitions of preventable harm

**Figure 2:**
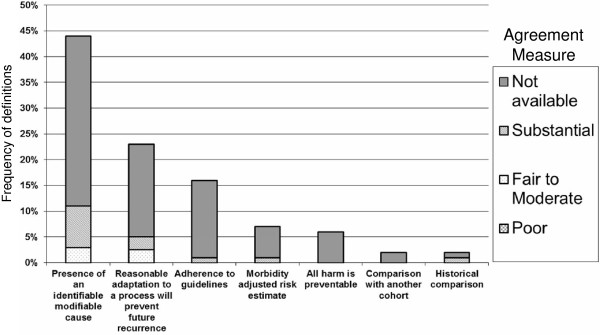
Frequency of definitions of preventable harm and the level of agreement reported in the studies that used multiple evaluators to assign a preventability level.

Each retrieved publication provided a single definition with the exception of two [5,9] which respectively provided five and two definitions. A hundred and thirty two definitions were analyzed. These fell into seven themes for preventable harm (Figure 2):

1) Presence of an identifiable modifiable cause: 44% (58/132)

2) Reasonable adaptation to a process will prevent future recurrence: 23% (30/132)

3) Lack of adherence to guidelines implies preventability: 16% (22/132)

4) Morbidity adjusted risk estimates using observed over expected models to account for preventable vs. inevitable harm (models typically adjust for severity of illnesses, patient demographics, comorbid conditions, and diagnoses): 7% (9/132)

5) All harm is preventable: 6% (8/132),

6) Comparison with another cohort shows different incidence: 2% (3/132)

7) Historical comparison– events with declining incidence over time are considered to be preventable in current practice: 2% (2/132)

**Figure 3:**
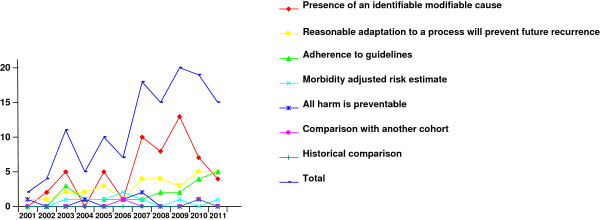
Year of publication.

There was an increase in the number of publications in the second half of the last decade. This was accounted for by the increased use of mainly two definitions: presence of an identifiable modifiable cause and adherence to guidelines (Figure 3).

Most of these definitions were author derived (67/127, 53%) and did not cite a specific source of the definition of preventable harm. In the remaining articles, three publications were cited the most as a source for the definition of preventability [10-12]. Notably, the definition used in these three articles was “the presence of an identifiable modifiable cause”.

### Severity of harm

Fifteen of the included studies provided a description of the severity of harm reported. It was mostly described when the harm was an adverse drug event (in 7 studies). Three of the studies considered only severe harm and 12 included all levels of harm. Fifty percent of the studies that provided specific severity classification used the “Presence of an identifiable modifiable cause” as a definition. Unfortunately, data were sparse and insufficient to analyze the severity of harm and its effect on preventability.

### Validity and operational characteristics

We found no data on the validity or feasibility of using the various definitions. Several studies however, reported a measure of agreement (often reported as a kappa statistic) among the researchers of the study. This measure described their agreement on the assignment of preventability of the harms reported in their study (Figure 2). Across all studies and all definitions, kappa statistic ranged from 0.23 to 0.95. Mean kappa statistic reported in the studies for each definition domain was: 0.82 (reasonable adaptation to a process will prevent future recurrence), 0.76 (morbidity adjusted risk estimate), 0.64 (presence of an identifiable modifiable cause), 0.62 (historical comparison) and 0.54 (adherence to guidelines). No agreement data was available for the remaining definitions.

## Discussion

### Main findings

Reducing patient harm has been identified as one of the main areas needed to improve both outcomes and costs of healthcare. The US Department of Health and Human Services, in forming the Partnership with Patients initiative has specifically targeted a reduction in preventable harm by 40% as one of its two key goals [13]. The American Food and Drug Administration’s Safe Use Initiative has targeted to “measurably reduce preventable harm from medications [14].” Other international agencies and organizations are also highly interested in this area. To measure our progress toward these goals, we need a validated metric that clearly incorporates the definition of a medical harm event and the level of preventability of such an event. Consulting the literature using a structured search strategy, we have identified seven key “themes” in extant definitions of “preventable harm”, with the most common being a) a harm with an identifiable and modifiable cause, b) “harm where a reasonable adaptation to a process will prevent future recurrence”, and c) “harm where an existing guideline has not been adhered to”. Our review revealed that, for the most part, these definitions are locally derived (67/127, 53%) and lack external validation. Interestingly, some of the other definitions such as all harm is preventable, were less commonly used. This implies that the definition of a Never Event is somewhat vague, dynamic and not standardized. Indeed, the 25 Never Events defined by the UK National Patient Safety Agency have numerous exceptions and modifications that highlight this challenge. We will discuss the three most common definition domains encountered in more detail and provide suggestions on what we think is a suitable approach to advance this critical area of health care delivery.

1) We have shown that the most common theme used to define preventability was “a harm with an identifiable and modifiable cause.” In studies using this definition, the researchers apparently had confidence that they could identify a specific “cause” and conclude that it was preventable because there was a single correct and achievable management choice (e.g., one medication was ordered but another delivered, medication error), or because the raters determined that most practitioners would have followed a different (more desirable) management plan. Such criteria may risk becoming circular to some extent; a preventable cause is preventable. At a minimum, to minimize bias and inaccuracy, one would hope for assurance of the reproducibility and agreement of such judgments between observers (typically measured with a chance-adjusted measure such as the kappa statistic). Other characteristics such as criterion and convergent validity of the measure will be desirable but hard to achieve.The major threat to the validity of such a judgment is hindsight bias. To avoid this situation and preserve the validity of this definition, the direct cause of harm should be definitely detectable before it has an opportunity to cause harm (e.g., administering the wrong drug to the patient). Some studies attempted to prevent hindsight bias (e.g., Hayward et al [7]. gave instructions to reviewers of harm cases to not consider certain aspect of the patient history and process of care); however, this is always challenging in observational studies. This definition and protection from hindsight bias requires an all-or-nothing cause-and-effect relationship, the kind associated with large effects. For causes weakly associated with harm (e.g., low quality handoffs among trainees), studies with rigorous protection against bias (e.g., randomized trials) would be needed to establish that harm is indeed preventable by affecting a specific causal pathway (e.g., improved communication tools to enhance resident handoff). One challenge in using this definition is that it does not always include “near misses”. These are cases in which harm did not occur but was likely to take place.Medication events were the most common type of harm described with this theme (“a harm with an identifiable and modifiable cause”) (20 out of 58 articles), and for those, only 7/20 had agreement measures reported, with the median kappa = 0.69 (range 0.49-0.93); consistent with good agreement. We have no data regarding the agreement from the other 13 studies examining medications that embraced this definition for preventable harm. One might suspect it would be harder to assign preventability to non-medication events where the process of care is more complex and less explicitly defined than it would be for adverse medication events, where pharmaceutical prescription, transcription, distribution, and ultimately administration is typically well worked out. However, there were measures of agreement in 8/38 studies addressing more complex causal chains associated with harm under this definition, with a median kappa = 0.68 (0.23-0.81). Thus, using this definition, the processes used and developed locally lead to good agreement in determination of preventable harm when measured. However, it is important to recognize here that the included studies judged preventability a posteriori; thus, hindsight bias has clearly inflated the observed agreement. There were no assessments of criterion or convergent validity.

2) In some ways, the second most common definition (“harm where a reasonable adaptation to a process of care will prevent recurrence of harm”) is an extension of the most common definition reviewed above. Using this definition, the root cause(s) of harm were identified and the reviewer(s) judged that modifying the processes of care could prevent this type of cause from repeating itself under the same or similar circumstances. Here, evaluation of drug events made up only 8/30 such studies, with the remainder examining a wide variety of medical events. Only one evaluated agreement between observers (kappa = 0.95) [15]. For non-medication event studies using this definition, only two studies measured agreement with Cohen’s kappa ranging from 0.68 to 0.83, consistent with moderate to substantial agreement. In 20, there was no assessment of agreement, and again, there were no external assessment of validity or control for hindsight bias. Perhaps most clearly here, this definition would require rigorous designs to establish with confidence that a certain modification could certainly lead to harm avoidance.

3) The third most common definition of preventable harm was “harm where an existing guideline has not been adhered to”. This definition introduces slightly different criteria to the assessment of whether or not an event is determined to be preventable from the prior ones. It is assumed that the existing guideline applies to the given situation, and that if the guideline was followed correctly, any harm associated with care should be considered to be not preventable. Conversely, any harm occurring in a situation in which the guideline was not followed is credited as “preventable” even if there is the possibility for disconnection between cause and effect. It is a measure of the reliability of care associated with situations that can result in harm. Twenty studies used this criterion, and 4 reported agreement with median kappa = 0.57 (0.40-.99). Event types were mostly related to venous thromboembolism (4), healthcare associated infections (5), or adverse drug events (2); all events for which there are generally well-established care guidelines. It is not clear why there appeared to be less agreement (lower kappa) in these situations compared with other definition types. However, in several cases, the care guidelines used were externally developed (e.g., the ACCP guidelines for venous thromboembolism prophylaxis) [16], so this data might reflect some measure of criterion validity. Other definitions were not used as often and most did not have assessments of agreement. It is also important to recognize that a significant proportion of guidelines either have poor quality and lack rigor or are based on low quality evidence [17].

### The quality of evidence

As acknowledged in the reviewed literature, most of measures of preventable harm were not created for comparison purposes; they were locally created to foster engagement of the local caregivers in efforts to improve patient safety. Measures based on administrative data, though less “subjective” and hence more applicable for cross-platform comparison, suffer from their own validity problems. First, such measures rely on administrative data, so that documentation and coding can improve “performance measures” more readily than actually *bona fide* improvement in safety and reliability. Secondly, administrative indices include adverse events that, by a reasonable standard with today’s science and technology, are not truly preventable. For instance, the skin organ system, like the liver organ system, at some point in the aging and critically ill patient may fail. Even with the best bedding materials, operative tables and conscientious nursing skills and diligent evidence-based care, some pressure ulcers are likely inevitable in contemporary practice. Failure to distinguish such events from those that may actually be preventable affects the credibility of the measure with clinicians, who may then dismiss other aspects of the measures.

An ideal metric would have characteristics of being accurate, valid, reproducible, and would have a high potential to engage caregivers in improvement work. As any other causal inference, the confidence in an estimate of a causal association between a modifiable event and the risk of harm is related to the validity of the evidence supporting such association. As in therapeutic associations, randomized trials are better suited to establish the preventable harm association when the risk of harm is moderate to high and the association small (when multiple contributing factors exist and noise and bias are of similar size as the signal or effect being detected). Large effects in a context where confounders are not problematic, compelling dose–response evidence, and protections against ascertainment bias of exposure and harm and selection bias further the quality of this evidence. All-or-nothing associations are associated with large and idiosyncratic harms and their link is obvious (e.g., wrong side surgery). However, the chain of events that led to such an event may be complex and convoluted and solutions to reduce the chance that this harmful process will repeat will likely require empirical evidence of efficacy. Given the known limitations of standards of care and guideline statements, their efficacy in preventing harm should also be empirically tested. Therefore, the overall quality of the current evidence in the field (strength of inference) remains quite limited.

### Principles for forging ahead

Preventable harm, therefore, appears to be best defined by three criteria: (a) harm with an incidence that can be reduced by virtue of detecting and intervening or preventing a causal event or chain of events (an error, an error-prone process, deviation from best practice), (b) the causal event or chain of events by their nature can be detected before the harm takes place, (c) there is evidence that an intervention is efficacious in reducing or eliminating the harm by virtue of eliminating the offending cause or disrupting a harmful chain of events. In addition, events to be included in assessment for preventability ought to have clear and validated criteria for harm level. As we have seen, the available definitions offer elements of the first one or two of these criteria with many affected by hindsight bias (violation of the second criterion). Few push their definitions to require empirical evidence of preventability.

Given the low level of confidence the data afford regarding measures of preventability, perhaps it would be prudent to add a modifier that would reflect the extent of adherence to these standards, such as definite, probable, and plausible. Definitely preventable harms, for instance, will apply to wrong treatment situations or error-prone complex processes with empirical evidence from a randomized trial that an intervention (e.g., checklist) consistently reduces the risk of harm by a large extent. Definitely preventable harms are ready to become targets for improvement and accountability. Probably and plausibly, preventable harms may require additional empirical work to become targets for improvement and accountability. As we discussed above, as the evidence evolves (new technologies appear, new evidence supporting these technologies emerges), harms which may have been plausibly preventable or not preventable may become definitely preventable. This classification therefore allows the safety community to work not only to reduce harm and improve safety but also to enhance the quality of the science of healthcare delivery. Future primary research in this area is needed to advance the field. Randomized or quasi randomized trials (e.g., pre-post design, comparative controlled cohort studies) will be needed to test the effectiveness of interventions that target harm associated with the delivery of healthcare. These trials will help determine a possible ceiling for improvement and define what is preventable. We believe that establishing a definition of preventable harm is desirable and may help guide quality improvement efforts and safety initiatives; however, a proposed definition would need to be sufficiently nuanced to reflect the setting and purpose of the preventability designation.

### Limitations

This systematic review has several limitations that are worth acknowledging. Judgments made by reviewers of the literature, despite the good inter-reviewer agreement, remain subjective. The definitions are clearly correlated and do not represent independent constructs. We focused our literature search on the last decade aiming at identifying a contemporary view; however, prior literature may provide relevant data. We searched using text-words in English which also may have led to omitting additional relevant information. The analysis in this systematic review was meta-narrative, i.e., non-quantitative; hence, providing more specific estimates for the operational characteristics of definitions was not feasible. Lastly, this review is focused on preventability and not ameliorability. Others have distinguished between preventable adverse drug reactions (caused by an error in management) and ameliorable (their severity could have been significantly reduced if health care delivery had been optimal) [18]. The ameliorability concept is not applicable to all harms (e.g., mortality) and the current systematic review does not address its definitions.

## Conclusions

There is limited empirical evidence of the validity and reliability of the available definitions of preventable harm, such that no single one is supported by high quality evidence. The most common definition is “presence of an identifiable, modifiable cause of harm”.

## Disclaimer

This systematic review adheres to the PRISMA guidelines.

## Competing interests

The authors declare that they have no competing interests.

## Authors’ contributions

MHM, MN, and VL designed the study. LP performed the search strategy and retrieved the data. MN, TBE, and MHM reviewed the articles and determined eligibility for inclusion, and drafted the manuscript. TM, JD, JN, AL, SS, VR, RT, and RT made substantial contributions to the analysis and interpretation of data, as well as, drafting of the manuscript. All authors approved the final manuscript.

## Pre-publication history

The pre-publication history for this paper can be accessed here:

http://www.biomedcentral.com/1472-6963/12/128/prepub

## Supplementary Material

Additional file 1 Actual search strategy. Click here for file

Additional file 2 **Table S1.**Characteristics of included publications. Click here for file

Additional file 3 ** Table S2.** Number of articles reported under each definition, broken up by the type of harm. Click here for file
