# Supplementary material for: What is preventable harm in healthcare? A systematic review of definitions
Source: BMC Health Serv Res. 2012 May 25;12:128. doi: 10.1186/1472-6963-12-128 (PMC3405467; doi:10.1186/1472-6963-12-128)
Supplement: Additional file 1 — Actual search strategy. [file 1472-6963-12-128-S1.doc]

Ovid

Database(s): Embase 1988 to 2011 Week 23, Ovid MEDLINE(R) In-Process & Other Non-Indexed Citations and Ovid MEDLINE(R) 1948 to Present
Search Strategy:

| **#** | **Searches** | **Results** |
| --- | --- | --- |
| 1 | preventable harm.mp. | 51 |
| 2 | preventable patient harm.mp. | 8 |
| 3 | 1 or 2 | 56 |
| 4 | exp Medical Errors/cl, mt, pc, st, sn [Classification, Methods, Prevention & Control, Standards, Statistics & Numerical Data] | 13393 |
| 5 | exp medical error/pc [Prevention] | 16692 |
| 6 | (preventable or avoidable or unnecessary).mp. | 91548 |
| 7 | (4 or 5) and 6 | 915 |
| 8 | 7 and (preventable or avoidable or unnecessary).ti. | 163 |
| 9 | exp Safety Management/cl, st, sn [Classification, Standards, Statistics & Numerical Data] | 1705 |
| 10 | exp Safety/st, sn [Standards, Statistics & Numerical Data] | 2596 |
| 11 | exp patient safety/ | 25712 |
| 12 | exp Patients/ | 555754 |
| 13 | (9 or 10) and 12 | 27 |
| 14 | (measur* or standard* or evidence-based or guideline*).mp. | 5514796 |
| 15 | (11 or 13) and 14 | 8022 |
| 16 | (measur* or standard* or evidence-based or guideline*).ti. and 15 | 850 |
| 17 | 16 and patient safety.ti. | 59 |
| 18 | ((measur* or standard* or evidence-based or guideline*) and patient safety).ti. | 389 |
| 19 | remove duplicates from 18 | 227 |
| 20 | (harm or complications).mp. [mp=ti, ab, sh, hw, tn, ot, dm, mf, dv, kw, ps, rs, nm, ui] | 1166181 |
| 21 | ((preventable or avoidable or unnecessary) adj2 (harm or complications)).mp. | 1141 |
| 22 | ((preventable or avoidable or unnecessary) adj2 (harm or complications)).ti. | 133 |
| 23 | 14 and 21 | 369 |
| 24 | (safety adj2 patient*).mp. [mp=ti, ab, sh, hw, tn, ot, dm, mf, dv, kw, ps, rs, nm, ui] | 71995 |
| 25 | 23 and 24 | 57 |
| 26 | 3 or 8 or 17 or 18 or 22 or 25 | 748 |
| 27 | ((define or defined or defining) adj3 "patient safety").mp. [mp=ti, ab, sh, hw, tn, ot, dm, mf, dv, kw, ps, rs, nm, ui] | 42 |
| 28 | 26 or 27 | 785 |
| 29 | pronovost pj.au. | 541 |
| 30 | 29 and safety.mp. [mp=ti, ab, sh, hw, tn, ot, dm, mf, dv, kw, ps, rs, nm, ui] | 226 |
| 31 | colantuoni e*.au. | 57 |
| 32 | safety.mp. and 31 [mp=ti, ab, sh, hw, tn, ot, dm, mf, dv, kw, ps, rs, nm, ui] | 12 |
| 33 | 28 or 30 or 32 | 1001 |
| 34 | remove duplicates from 33 | 632 |
| 35 | limit 34 to english language | 581 |
| 36 | limit 35 to yr="2001 -Current" | 532 |
| 37 | limit 36 to (letter or note or news) [Limit not valid in Embase,Ovid MEDLINE(R),Ovid MEDLINE(R) In-Process; records were retained] | 52 |
| 38 | 36 not 37 | 480 |
