# Supplementary material for: What is preventable harm in healthcare? A systematic review of definitions
Source: BMC Health Serv Res. 2012 May 25;12:128. doi: 10.1186/1472-6963-12-128 (PMC3405467; doi:10.1186/1472-6963-12-128)
Supplement: Additional file 2 — Table S1.Characteristics of included publications. [file 1472-6963-12-128-S2.doc]

Supplemental Table 1: Characteristics of included publications

| **Article label** | **Type** | **Objective** | **Population** | **setting** | **Source of Definition** | **How was "harm" defined** | **How was "preventable" defined** | **Sample size** | **Agreement/Kappa** | **Severity of reported harm** |
| --- | --- | --- | --- | --- | --- | --- | --- | --- | --- | --- |
| Arzy, 2009[^1^](#_ENREF_1) | Cross^a^ | To determine diagnostic accuracy in the presence of a single misleading detail | Attending doctors in internal medicine | Inpatient | Unclear | Diagnostic error | Presence of an identifiable modifiable cause | NA | NA | NA |
| Aspden, 2005[^2^](#_ENREF_2) | Opinion piece | To put forward a road map for the development and adoption of key health care data standards to support both information exchange and the reporting and analysis of patient safety data | NA | NA | Author-derived | Various | Presence of an identifiable modifiable cause | NA | NA | NA |
| Baker, 2004[^3^](#_ENREF_3) | R, Obs^b^ | To estimate  the incidence of adverse events (AEs) among patients in Canadian acute care hospitals | Hospitalized patients | Inpatient | Peer-reviewed citation | Various | Presence of an identifiable modifiable cause | 3745 charts from different types of Canadian hospitals | Kappa=0.69 | NA |
| Bapoje, 2011[^4^](#_ENREF_4) | R, Obs | To determine why unplanned transfers occur, what fraction results from errors in care, whether they are preceded by changes in clinical status and if so, whether earlier or different responses might prevent the transfers | Patients with unplanned transfers to the medical ICU | Inpatient | Author-derived | (Other) Incorrect triage at time of admission, iatrogenic errors | Reasonable adaptation to a process will prevent future recurrence | 152 ICU transfers (15% were preventable) | Kappa=0.68 | NA |
| Bartlett, 2008[^5^](#_ENREF_5) | Cross | To assess whether communication problems are associated with an increased risk of preventable adverse events | Hospitalized patients | Inpatient | Peer-reviewed citation | Adverse drug events | Reasonable adaptation to a process will prevent future recurrence | NA | NA | NA |
| Beckmann, 2003[^6^](#_ENREF_6) | Cross | To evaluate facilitated incident monitoring (FIM) and medical chart review (MCR) in the intensive care setting | ICU patients | Inpatient | Author-defined | Various (An adverse event was defined as an unintended injury or complication, which prolonged the hospital stay or led to death or disability at the time of discharge, that was caused by healthcare management rather than the patient’s disease) | Adherence to guidelines | 176 ICU admissions | 99% (Only 3 out of 256 incidents required judging) | NA |
| Berenholtz, 2007[^7^](#_ENREF_7) | Opinion piece | To describe a framework that health care organizations can use to monitor patient safety | Intensive care unit patients | Inpatient | Author-defined | Health care-associated infections | Reasonable adaptation to a process will prevent future recurrence | NA | NA | NA |
| Berenholtz, 2011[^8^](#_ENREF_8) | P, Obs^c^ | To evaluate the impact of a multifaceted intervention on compliance with evidence-based therapies and ventilator-associated pneumonia (VAP) rates | Intensive care unit patients | Inpatient | Consensus | Health care-associated infections | Adherence to guidelines | 112 ICU's | NA | NA |
| Brilli, 2010[^9^](#_ENREF_9) | Opinion piece | To describe a motivational tool, the preventable harm index, that was developed to facilitate the drive to reduce preventable harm at the author's hospital | Hospitalized patients | Inpatient | Author-derived | Various | All harm is preventable | NA | NA | NA |
| Brooke, 2010[^10^](#_ENREF_10) | Case-control | To evaluate whether implementation of Leapfrog’s standard for routine b-blockade was associated with reductions in mortality after open bdominal aortic aneurysm (AAA) repair alone versus other high-risk operations | Abdominal aortic aneurysm patients | Inpatient | Consensus | (Other) Peri-operative adverse events | Reasonable adaptation to a process will prevent future recurrence | NA | NA | NA |
| Buckley, 2007[^11^](#_ENREF_11) | P, Obs | To determine the incidence, type, and stage of occurrence of medication errors and potential and actual adverse drug events (ADEs) in a pediatric intensive care unit (ICU) | Children | Inpatient | Peer-reviewed citation | Adverse drug events | Presence of an identifiable modifiable cause | 58 incidents were evaluated | kappa=0.93 (prevalence-adjusted and bias-adjusted) | Variable severity included. Severity of events included was classified based on the works of Bates et al (1995). Potentially lethal or serious events were included. Particular attention was paid to medications labeled as “high alert” medications. |
| Burda, 2005[^12^](#_ENREF_12) | P, Obs | To examine the extent of medication and allergy discrepancies between surgical and anesthesia preoperative medication histories for patients admitted to two surgical intensive care units in an academic medical center | Surgical patients | Inpatient | Author-derived | (Other) Unclear | Reasonable adaptation to a process will prevent future recurrence | NA | NA | NA |
| Canale, 2005[^13^](#_ENREF_13) | Opinion piece | To emphasize the importance of the Sign Your Site protocol | Surgical patients | NA | Peer-reviewed citation | Wrong-site surgery | Adherence to guidelines | NA | NA | NA |
| Carpenter, 2010[^14^](#_ENREF_14) | SR | To review the literature on patient safety issues in developing and emerging countries | Hospitalized patients | Inpatient | Peer-reviewed citation | Various | Presence of an identifiable modifiable cause | NA | NA | NA |
| Catalano, 2008[^15^](#_ENREF_15) | Opinion piece | NR | Hospitalized patients | Inpatient | Peer-reviewed citation | (Other) Hospital-acquired conditions | All harm is preventable | NA | NA | NA |
| Chang, 2008[^16^](#_ENREF_16) | R, Obs | To determine the pattern of Patient Safety Indicators among adult trauma patients | Adult trauma patients | Inpatient | Consensus | Various | Presence of an identifiable modifiable cause | NA | NA | NA |
| Chaung, 2007[^17^](#_ENREF_17) | Opinion piece | To address the gap between awareness of preventable adverse events and knowledge that relates to how to respond to them effectively | Universal | All settings | Unclear | Various | Reasonable adaptation to a process will prevent future recurrence | NA | NA | NA |
| Cohen, 2008[^18^](#_ENREF_18) | Cross | To assess the prevalence of VTE risk in the acute hospital care setting, and to determine the proportion of at-risk patients who receive effective prophylaxis | Hospitalized patients | Inpatient | Peer-reviewed citation | Hospital-stay related venous thromboembolism | Adherence to guidelines | NA | NA | NA |
| Cooper, 2002[^19^](#_ENREF_19) | R, Obs | To uncover patterns of frequently occurring incidents that are in need of careful prospective investigation | Staff and resident anesthesiologists | Inpatient | Author-derived | Various | Presence of an identifiable modifiable cause, Adherence to guidelines | NA | NA | NA |
| Cupryk, 2011[^20^](#_ENREF_20) | Opinion piece | To present the challenges in patient safety risk management for the patient, US Food and Drug Administration, health care provider, and sponsor | NA | NA | Consensus | Various | Presence of an identifiable modifiable cause | NA | NA | NA |
| Dagi, 2007[^21^](#_ENREF_21) | Opinion piece | To report on preventable operating room errors and strategies to avoid them | Surgeons and OR scrub personnel | Inpatient | Author-defined | (Other) Retained foreign objects, sharps injuries, and wrong site surgery | Reasonable adaptation to a process will prevent future recurrence | NA | NA | NA |
| Daniels, 2010[^22^](#_ENREF_22) | Cross | To develop a web-based system, the Family Reporting System (FRS), to elicit adverse event reports from families of children admitted to hospital through survey methodology and human factors engineering techniques | Parents and\or guardians of admitted children | Inpatient | Peer-reviewed citation | Various | Reasonable adaptation to a process will prevent future recurrence | 285 reports were completed | 83% agreement | All degrees of harm were evaluated |
| Davis, 2003[^23^](#_ENREF_23) | Cross | To describe the pattern of preventable in-hospital medical injury under the “no fault” system and to assess the level of serious preventable patient harm | Hospitalized patients | Inpatient | Author-defined | Various | Adherence to guidelines | 6579 patients in 13 hospitals | 87.5% agreement (kappa 0.47) | Serious impact  of an adverse event was defined as permanent disability (lasting  more than 1 year) or death |
| De Wet, 2011[^24^](#_ENREF_24) | Consensus statement | To describe how to apply a recently developed trigger tool for primary care | Patients in primary care | Outpatient | Consensus | Various | Reasonable adaptation to a process will prevent future recurrence | NA | NA | NA |
| DePalo, 2010[^25^](#_ENREF_25) | P, Obs | To describe the Rhode Island ICU collaborative to reduce CLABSI and VAP | ICU patients | Inpatient | Author-defined | CLABSI and VAP | Reasonable adaptation to a process will prevent future recurrence | NA | NA | NA |
| Devine, 2010[^26^](#_ENREF_26) | P, Obs | To evaluate the effect of a basic, ambulatory Computerized Provider Order Entry (CPOE) system on medication errors and associated adverse drug events ADEs | Outpatients | Outpatient | Consensus | Adverse drug events | Historical comparison | Over 10,000 prescriptions reviewed (Before and after the implementation of EHRs) | kappa=0.62 (93% agreement) | NA |
| Dupuis, 2007[^27^](#_ENREF_27) | R, Obs | To determine the frequency of avoidable neonatal neurological damage | Neonates | Inpatient | Author-defined | (Other) Neonatal neurological damage | Presence of an identifiable modifiable cause | NA | NA | NA |
| Elder, 2002[^28^](#_ENREF_28) | SR | To describe and classify process errors and preventable adverse events that occur from medical care in outpatient primary care settings | Outpatients | Outpatient | Peer-reviewed citation | Various | Presence of an identifiable modifiable cause | NA | NA | NA |
| Friedley, 2008[^29^](#_ENREF_29) | Opinion piece | Health and financial implications of adverse drug events | Patients at a primary care office | Outpatient | Consensus | Adverse drug events | Presence of an identifiable modifiable cause | NA | NA | NA |
| Friedman, 2008[^30^](#_ENREF_30) | R, Obs | To analyze data from the multinational Global Orthopedic Registry (GLORY) to evaluate the compliance of surgeons with the American College of Chest Physicians (ACCP) guidelines for VTE prevention | Hospitalized patients | Inpatient | Peer-reviewed citation | Hospital-stay related venous thromboembolism | Adherence to guidelines | NA | NA | NA |
| Garner, 2001[^31^](#_ENREF_31) | Opinion piece | To address the issue of medication errors in the elderly | Nursing home residents | Inpatient | Peer-reviewed citation | Adverse drug events | All harm is preventable | NA | NA | Variable severity included. 94% of ADE’s included were significant/serious. The rest were fatal(1 event)/life-threatening. |
| Gibbs, 2007[^32^](#_ENREF_32) | Opinion piece | To report on preventable operating room errors and strategies to avoid them | Surgical patients | Inpatient | Author-defined | (Other) Retained foreign objects | Presence of an identifiable modifiable cause | NA | NA | NA |
| Gilbert, 2009[^33^](#_ENREF_33) | Opinion piece | Infection control, ethics and accountability | Hospitalized patients | Inpatient | Peer-reviewed citation | Health care-associated infections | Adherence to guidelines | NA | NA | NA |
| Greene, 2004[^34^](#_ENREF_34) | Cross | To determine how good medical staff are in estimating patients body weights | Medical staff | Inpatient | Author-defined | Adverse drug events | Reasonable adaptation to a process will prevent future recurrence | NA | NA | NA |
| Gruen, 2006[^35^](#_ENREF_35) | R, Obs | To identify patterns of errors contributing to inpatient trauma deaths | Trauma patients | Inpatient | Peer-reviewed citation | Death | Adherence to guidelines | NA | NA | Focused on errors that were associated with the most serious consequence, death. |
| Gurses, 2010[^36^](#_ENREF_36) | Opinion piece | To identify factors that affect clinicians’ compliance with the evidence-based guidelines | Hospitalized patients | Inpatient | Peer-reviewed citation | Health care-associated infections | Adherence to guidelines | NA | NA | NA |
| Halfon, 2007[^37^](#_ENREF_37) | R, Obs | To develop a screening algorithm for avoidable reoperations using only routinely collected hospital data and a prediction model to adjust rates for case-mix | Post-op hospitalized patients | Inpatient | Author-defined | (Other) Avoidable reoperation | Morbidity adjusted risk estimate | 833 reoperations were reviewed | Kappa=0.76 | NA |
| Haller, 2008[^38^](#_ENREF_38) | R, Obs | To assess the value of UIA as a global measure of avoidable iatrogenic complications in surgical patients | Surgical patients | Inpatient | Author-defined | (Other) Unplanned post-op ICU admission | Presence of an identifiable modifiable cause | 188 unplanned ICU admissions were reviewed | Agreement was 79.7%, with a kappa=0.23 | NA |
| Heitmiller, 2007[^39^](#_ENREF_39) | Opinion piece | To assess the validity of anesthesia-related death claims as a measure of Incidence of patient harm | Anesthesia-related deaths | Inpatient | Author-defined | Various | Reasonable adaptation to a process will prevent future recurrence | NA | NA | NA |
| Hofer, 2002[^40^](#_ENREF_40) | Opinion piece | To explore the issue of bad outcome from questionable clinical decisions as a preventable medical error | Hospitalized patients | Inpatient | Peer-reviewed citation | (Other) An injury or complication caused by medical management that prolongs hospital stay or produces lasting disability or death | Presence of an identifiable modifiable cause | NA | NA | NA |
| Hoff, 2011[^41^](#_ENREF_41) | SR | To examine over an initial 3-year period academic and trade articles addressing the Centers fot Medicare and Medicaid Services (CMS) policy to gain the impressions, guidance, and content provided in this literature | Hospitalized patients | Inpatient | Consensus | Various | Presence of an identifiable modifiable cause | NA | NA | NA |
| Holzmueller, 2005[^42^](#_ENREF_42) | Opinion piece | To develop an incident reporting system that caregivers in a diverse group of ICUs would use; to identify factors contributing to incidents from a systems perspective; and to disseminate lessons learned in an effort to broadly improve safety | ICU staff | Inpatient | Author-defined | Various | Reasonable adaptation to a process will prevent future recurrence | NA | NA | NA |
| Hoonhout, 2010[^43^](#_ENREF_43) | R, Obs | To provide information on the nature, consequences and preventability of medication -related adverse events (MRAEs) occurring during hospitalization in the Netherlands | Hospitalized patients | Inpatient | Author-defined | Adverse drug events | Adherence to guidelines | 148 medication related adverse events in 140 hospital admissions were reviewed | Agreement was 70% with a kappa=0.40 | NA |
| Howard, 2003[^44^](#_ENREF_44) | R, Obs | To describe the drugs and types of medicine management problems most frequently associated with preventable drug related admissions to an acute medical admissions unit | Patients admitted for drug related events | Inpatient | Author-defined | Adverse drug events | Presence of an identifiable modifiable cause | Over 4000 patients seen by pharmacists on the medical admissions unit | kappa=0.74 | NA |
| Hug, 2009[^45^](#_ENREF_45) | R, Obs | To assess the incidence and severity of adverse drug events (ADEs) in patients with reduced creatinine clearance | Patients with reduced creatinine clearance | Inpatient | Author-defined | Adverse drug events | Presence of an identifiable modifiable cause | Over 109,000 admissions in 6 study sites were reviewed | 95.1% agreement for preventability (kappa=0.64) | Variable severity included. Evaluation of incidents by severity showed that most were  Serious (51.1%) or significant  (44.4%). Of ADEs  4.5% were life threatening (0.44/100 admissions) |
| Hughes, 2006[^46^](#_ENREF_46) | Opinion piece | To describe the development of Potentially Preventable Complications (PPCs), a new method that uses a present on admission (POA) indicator to identify in-hospital complications among secondary diagnoses that arise after admission | Hospitalized patients | Inpatient | Author-defined | Various | Morbidity adjusted risk estimate | NA | NA | NA |
| Ibrahim, 2009[^47^](#_ENREF_47) | Opinion piece | To describe how the advent of patient safety is changing the traditional ‘hospital mortality audit" |  | Inpatient | Author-defined | Death | Morbidity adjusted risk estimate | NA | NA | NA |
| Iyengar, 2009[^48^](#_ENREF_48) | P, Obs | To describe how Medical Emergency teams (MET) calls were used to systematically identify preventable adverse events in an academic tertiary care hospital | MET refferrals | Inpatient | Consensus | Various | Reasonable adaptation to a process will prevent future recurrence | NA | NA | Variable severity included. 19% of patients with preventable adverse events were considered  life-threatening |
| Jennings, 2008[^49^](#_ENREF_49) | P, Obs | To demonstrate formal evaluations of anti-coagulant use practices and associated patient outcome | Hospitalized patients | Inpatient | Author-defined | Adverse drug events | Reasonable adaptation to a process will prevent future recurrence | NA | NA | NA |
| Kaissi, 2003[^50^](#_ENREF_50) | Cross | To measure the attitudes and team work related to patient safety concerns in high-risk areas including the OR, ED, and ICU are measured | Staff of high-risk areas (OR, ED, ICU) | Inpatient | Unclear | Various | Reasonable adaptation to a process will prevent future recurrence | NA | NA | NA |
| Kanjanarat, 2003[^51^](#_ENREF_51) | SR | To identify the drug classes, types of errors, and types of adverse outcomes related to preventable adverse drug events (pADEs) | Hospitalized patients | Inpatient | Author-defined | Adverse drug events | Reasonable adaptation to a process will prevent future recurrence | NA | NA | NA |
| Karsh, 2010[^52^](#_ENREF_52) | Opinion piece | To discuss the impact of levels on theory, measurement, analysis and intervention in patient safety research | NA | NA | Unclear | Adverse drug events | Reasonable adaptation to a process will prevent future recurrence | NA | NA | NA |
| Kleinpell, 2009[^53^](#_ENREF_53) | Cross | To explore the relationship between the proportion of certified staff nurses in a unit and risk of harm to patients | Nurses | Inpatient | Author-defined | Various | Presence of an identifiable modifiable cause | NA | NA | NA |
| Kucukarslan, 2003[^54^](#_ENREF_54) | RCT | To evaluate the impact of having a pharmacist participate with a physician rounding team on preventable ADEs in general medicine units and to document pharmacist interventions made during the rounding process | Hospitalized patients | Inpatient | Author-defined | Adverse drug events | Presence of an identifiable modifiable cause | NA | Kappa = 0.79 | NA |
| Kunac, 2008[^55^](#_ENREF_55) | P, Obs | To evaluate the frequency and characteristics of preventable medication-related events in hospitalized children | Hospitalized children | Inpatient | Consensus | Adverse drug events | Presence of an identifiable modifiable cause | Over 3100 medication orders were reviewed | Excellent | NA |
| Kuzel, 2004[^56^](#_ENREF_56) | Cross | To develop patient-focused typologies of medical errors and harms in primary care settings and to discern which medical errors and harms seem to be the most important | Patients reporting incidents of harm | Outpatient | Author-defined | (Other) Breakdowns in access to and relationships with clinicians | All harm is preventable | NA | NA | NA |
| Laditka, 2006[^57^](#_ENREF_57) | Cross | To examine race and ethnicity differences in accessibility and effectiveness of health care during pregnancy | Pregnant Medicaid beneficiaries in South Carolina | Inpatient | Author-defined | (Other) Maternity complications | Reasonable adaptation to a process will prevent future recurrence | NA | NA | NA |
| Larsen, 2007[^58^](#_ENREF_58) | R, Obs | To develop a trigger tool for identifying adverse events occurring in critically ill pediatric patients; to identify and characterize adverse events and preventable adverse events experienced by critically ill pediatric patients; and to characterize the patients who experience preventable adverse events | Critically ill pediatric patients | Inpatient | Author-defined | Various | Presence of an identifiable modifiable cause | 259 PICU admissions were sampled from over 1800 admissions over a 1-year period | Interrater agreement was 86% (kappa=0.68) | Variable severity included (78% minor, 19% moderate, 3% serious, no  deaths) |
| Lee, 2009[^59^](#_ENREF_59) | P, Obs | To describe the epidemiology of controlled substance prescription errors by physicians-in-training for children being discharged from the hospital | Children being discharged from the hospital | Inpatient | Author-defined | Adverse drug events | Presence of an identifiable modifiable cause | NA | NA | Variable severity included. Prescription errors were graded between 1 (insignificant) and 5 (severe) |
| Lessing, 2010[^60^](#_ENREF_60) | SR | To perform a systematic review of the frequency of (preventable) adverse events (AE/PAE) | NA | All settings | Peer-reviewed citation | Various | Presence of an identifiable modifiable cause | NA | NA | NA |
| Lilly, 2011[^61^](#_ENREF_61) | P, Obs | To quantify the association of a tele-ICU intervention with hospital mortality, length of stay, and complications that are preventable by adherence to best practices | Intensive care unit patients | Inpatient | Author-defined | Various | Morbidity adjusted risk estimate | NA | NA | Variable severity included. 76% of the events were classified as major (eg, requiring  initiation of a vasoactive medication),  17% intermediate (eg, evaluation of an arrhythmia), and 7% as minor (eg, electrolyte correction). |
| Lu, 2006[^62^](#_ENREF_62) | R, Obs | To investigate the rate and types of preventable deaths among patients with early mortality after emergency admission from the ED | Patients admitted to the emergency department | Inpatient | Author-defined | Death | Presence of an identifiable modifiable cause | 210 early mortality cases were reviewed | Kappa=0.81 | NA |
| Mackinnon, 2003[^63^](#_ENREF_63) | R, Obs | To determine the incidence of preventable drug-related morbidity (PDRM) in older adults in a provider-sponsored network and identify risk factors for PDRM | Senior patients | Inpatient | Peer-reviewed citation | Adverse drug events | Morbidity adjusted risk estimate | NA | NA | NA |
| Makary, 2007[^64^](#_ENREF_64) | Cross | To evaluate the impact of operating room briefings on coordination of care and risk for wrong-site surgery | Operating room staff | Inpatient | Author-defined | Wrong-site surgery | Presence of an identifiable modifiable cause | NA | NA | NA |
| McBride, 2005[^65^](#_ENREF_65) | P, Obs | To determine the incidence of preventable adverse events (AEs) and near misses (NMs) among infants hospitalized for bronchiolitis | Infants hospitalized for bronchiolitis | Inpatient | Author-defined | (Other) Any injury resulting from medical management | Presence of an identifiable modifiable cause | 143 infants with bronchiolitis were studied | Kappa=0.71 | Variable severity included. The severity of each incident was rated as fatal, life threatening, severe, significant, or not injurious or harmful |
| McCulloch, 2010[^66^](#_ENREF_66) | Opinion piece | How to improve performance in the operating room by avoiding preventable harm | Patients undergoing surgery | Inpatient | Author-defined | (Other) Morbidity and mortality incurred from cancer curative surgery | Presence of an identifiable modifiable cause | NA | NA | NA |
| Michaels, 2007[^67^](#_ENREF_67) | SR | Review the evidence regarding methods to prevent wrong site operations | Surgical patients | Inpatient | Author-defined | Wrong-site surgery | All harm is preventable | NA | NA | NA |
| Michel, 2004[^68^](#_ENREF_68) | R, Obs; Cross; P Obs. | To compare the effectiveness, reliability, and acceptability of estimating rates of adverse events and rates of preventable adverse events using three methods: cross sectional, prospective, and retrospective | Hospitalized patients | Inpatient | Peer-reviewed citation | (Other) An unintended injury caused by medical management rather than by a disease process and which resulted in death, life threatening illness, disability at time of discharge, admission to hospital, or prolongation of hospital stay | Adherence to guidelines | 37 wards in seven hospitals were studied | Agreement on preventability was 67.8% (Kappa=0.31) | NA |
| Miller, 2007[^69^](#_ENREF_69) | SR | To synthesize peer reviewed knowledge on children’s medication errors and on recommendations to improve paediatric medication safety by a systematic literature review | NA | All settings | Peer-reviewed citation | Adverse drug events | Presence of an identifiable modifiable cause | NA | NA | 11 studies categorized medication errors by severity of outcome for the patient. At least four different scales were used to rank error severity. |
| Millman, 2011[^70^](#_ENREF_70) | case report | advocate for patient-incident reporting system | debilitated hospitalized patient with malignancy | Inpatient | Author-defined | (Other) Fall | Presence of an identifiable modifiable cause | NA | NA | NA |
| Morriss, 2009[^71^](#_ENREF_71) | P, Obs | To show the impact of using a barcode medication administration (BCMA) system on preventable adverse drug events (ADEs) in a neonatal intensive care unit | Neonates | Inpatient | Author-defined | Adverse drug events | Reasonable adaptation to a process will prevent future recurrence | NA | NA | Variable severity included. A severity level was assigned each preventable ADE  using the National Coordinating Council for Medication  Error Reporting and Prevention Index |
| Murphy, 2010, AJMQ[^72^](#_ENREF_72) | Cross | To assess statewide efforts to monitor and prevent CLABSI's | ICU patients | Inpatient | Peer-reviewed citation | CLABSI | comparison with another cohort | NA | NA | NA |
| Murphy, 2010, AMA[^73^](#_ENREF_73) | Opinion piece | address the incidence and preventability of health care-associated sepsis and pneumonia | Hospitalized patients | Inpatient | Author-defined | CLABSI and VAP | Presence of an identifiable modifiable cause | NA | NA | NA |
| Naessens, 2009[^74^](#_ENREF_74) | Cross | To determine the degree of congruence between several measures of adverse events | Inpatients | Inpatient | Peer-reviewed citation | Various | Presence of an identifiable modifiable cause | NA | NA | NA |
| Needham, 2004[^75^](#_ENREF_75) | Cross | To evaluate the contributing and limiting factors for airway events reported in the Intensive Care Unit Safety Reporting System (ICUSRS) developed in partnership with the Society of Critical Care Medicine | ICU patients | Inpatient | Author-defined | (Other) Airway events | Morbidity adjusted risk estimate | NA | NA | NA |
| Needham, 2005[^76^](#_ENREF_76) | Cross | To analyze the system factors related to “line, tube, and drain” (LTD) incidents in the ICU | ICU patients | Inpatient | Author-defined | (Other) Airway events | Morbidity adjusted risk estimate | NA | NA | NA |
| Newman, 2009[^77^](#_ENREF_77) | Opinion piece | To offer definitions for diagnostic error and misdiagnosis-related harm, and to present an overview of the magnitude of diagnostic errors, and give suggestions for how research can mature | NA | NA | Author-defined | Diagnostic error | Presence of an identifiable modifiable cause | NA | NA | NA |
| Newton, 2010[^78^](#_ENREF_78) | Consensus statement | To define the measures being taken to reduce the risks of using equipment in medicine | NA | NA | Consensus | (Other) Device failure leading to patient harm | Presence of an identifiable modifiable cause | NA | NA | NA |
| O'Grady, 2003[^79^](#_ENREF_79) | Opinion piece | To address the issue of catheter-related infections and the guidelines to prevent it | NA | NA | Consensus | CLABSI | Adherence to guidelines | NA | NA | NA |
| Pandey, 2007[^80^](#_ENREF_80) | P, Obs | To identify errors that took place in phlebotomy area, analyzing and classifying them | Blood donors | Inpatient | Consensus | (Other) Errors in the phlebotomy area | All harm is preventable | NA | NA | NA |
| Peterson, 2001[^81^](#_ENREF_81) | Opinion piece | Identifying and eliminating serious drug interactions as preventable medication errors | Hospitalized patients | Inpatient | Peer-reviewed citation | Adverse drug events | Reasonable adaptation to a process will prevent future recurrence | NA | NA | NA |
| Pham, 2011[^82^](#_ENREF_82) | Cross | To examine the frequency, types, causes, and consequences of voluntarily reported ED medication errors in the United States | Emergency department staff | Inpatient | Consensus | Adverse drug events | Adherence to guidelines | NA | NA | NA |
| Prescrire International_2007[^83^](#_ENREF_83) | Opinion piece | Preventing adverse effects of the SSI BCG vaccine | Patients taking the SSI BCG vaccine | NA | Author-defined | Adverse drug events | Presence of an identifiable modifiable cause | NA | NA | NA |
| Prescrire International_2007[^84^](#_ENREF_84) | Opinion piece | Preventing avoidable errors in Methotrexate therapy | Patients taking Methotrexate | NA | Author-defined | Adverse drug events | Presence of an identifiable modifiable cause | NA | NA | NA |
| Pronovost, 2004[^85^](#_ENREF_85) | Opinion piece | To provide a practical framework for improving patient safety | ICU patients | Inpatient | Consensus | CLABSI | Reasonable adaptation to a process will prevent future recurrence | NA | NA | NA |
| Pronovost, 2005[^86^](#_ENREF_86) | Opinion piece | To review the definition of safety and error and discuss approaches to measuring safety | NA | NA | Consensus | (Other) An injury resulting from a medical intervention | Reasonable adaptation to a process will prevent future recurrence | NA | NA | NA |
| Pronovost, 2006[^87^](#_ENREF_87) | P, Obs | To evaluate the frequency and type of factors involved in incidents reported to a patient safety reporting system and answer specific questions to enhance the value of PSRS data to improve patient safety | ICU patients | Inpatient | Author-defined | Various | comparison with another cohort | NA | NA | NA |
| Pronovost, 2009[^88^](#_ENREF_88) | Opinion piece | To provide strategies to differentiate between inevitable and preventable harm | NA | NA | Author-defined | Various | Comparison with another cohort, Historical comparison, Morbidity adjusted risk estimate, All harm is preventable, Adherence to guidelines, | NA | NA | NA |
| Pronovost, 2010, Anesth[^89^](#_ENREF_89) | Opinion piece | A lecture on patient safety and quality of care | Patients in ICU | Inpatient | Author-defined | Various | Reasonable adaptation to a process will prevent future recurrence | NA | NA | NA |
| Pronovost, 2010, BMJ[^90^](#_ENREF_90) | P, Obs | To evaluate the extent to which intensive care units participating in the initial Keystone ICU project sustained reductions in rates of catheter related bloodstream infections | Patients in ICU | Inpatient | Author-defined | CLABSI | Adherence to guidelines | NA | NA | NA |
| Pronovost, 2011[^91^](#_ENREF_91) | Consensus statement | To outline a 5-phase translational framework to develop robust research programs that reduce preventable harm | NA | NA | Consensus | Various | Reasonable adaptation to a process will prevent future recurrence | NA | NA | NA |
| Rabinowitz, 2008[^92^](#_ENREF_92) | Opinion piece | To discuss solutions aimed at reducing hospital medication errors | NA | NA | Consensus | Adverse drug events | Presence of an identifiable modifiable cause | NA | NA | NA |
| Rahme, 2008[^93^](#_ENREF_93) | R, Obs | To identify hospital and patient characteristics associated with receiving thromboprophylaxis after discharge and to compare the risk of short-term mortality among those who did or did not receive thromboprophylaxis | Hospitalized patients | Inpatient | Author-defined | Hospital-stay related venous thromboembolism | Reasonable adaptation to a process will prevent future recurrence | NA | NA | NA |
| Robbins, 2011[^94^](#_ENREF_94) | Opinion piece | Transforming evidence-based care and patient safety protocols into routine practice; surgical chicklists as an example | Surgical staff | Inpatient | Consensus | Various | Adherence to guidelines | NA | NA | NA |
| Rothschild, 2005[^95^](#_ENREF_95) | P, Obs | To highlight the incidence and nature of adverse events and serious medical errors in intensive care | ICU patients | Inpatient | Author-defined | Various | Presence of an identifiable modifiable cause | NA | NA | NA |
| Sadri, 2010[^96^](#_ENREF_96) | Cross | To check orthopedic surgery staff's understanding of tourniquete use | Orthopedic surgeons and staff | Inpatient | Author-defined | (Other) Avascular necrosis of the extremity | Presence of an identifiable modifiable cause | NA | NA | NA |
| Sawyer, 2010[^97^](#_ENREF_97) | Opinion piece | The use of a quality improvement program to eliminate CLABSI | ICU patients | Inpatient | Peer-reviewed citation | CLABSI | Presence of an identifiable modifiable cause | NA | NA | NA |
| Schlack, 2010[^98^](#_ENREF_98) | Opinion piece | To review the scientific basis of the WHO recommendations to improve patient safety during surgical procedures by using the Surgical Safety Checklist | Surgical staff | Inpatient | Consensus | Various | Adherence to guidelines | NA | NA | NA |
| Segal, 2011[^99^](#_ENREF_99) | Opinion piece | To identify avoidable errors in dealing with anaphylactoid reactions to iodinated contrast media | Patients undergoing diagnostic imaging | All settings | Author-defined | (Other) Anaphylactoid reaction | Presence of an identifiable modifiable cause | NA | NA | NA |
| Seiden, 2006[^100^](#_ENREF_100) | Cross | Search for wrong-side/wrong-site, wrong-procedure, and wrong-patient adverse events (WSPEs) in 4 different data-bases | Surgical patients | Inpatient | Consensus | Wrong-site surgery | All harm is preventable | NA | NA | NA |
| Selby, 2011 (In press)[^101^](#_ENREF_101) | R, Obs | To compare venous thromboembolism and bleeding event rates in patients adhereing to ACCP-recommended thromboprophylaxis and those who didn't | Hospitalized patients | Inpatient | Author-defined | Hospital-stay related venous thromboembolism | Adherence to guidelines | NA | NA | NA |
| Seliger, 2008[^102^](#_ENREF_102) | Cross | A Cross analysis of CKD and adverse safety events in hospitalized veterans | Hospitalized patients with CKD | Inpatient | Author-defined | Various | Presence of an identifiable modifiable cause | NA | NA | NA |
| Sexton, 2011[^103^](#_ENREF_103) | P, Obs | To evaluate the impact of a comprehensive unit based safety program on safety climate in a large cohort of intensive care units participating in the Keystone intensive care unit project | ICU staff | Inpatient | Author-defined | Various | Adherence to guidelines | NA | NA | NA |
| Sherman, 2008[^104^](#_ENREF_104) | Opinion piece | Treating a close relative considered a new type of medical error | NA | NA | Author-defined | (Other) Ttreating a close relative | Presence of an identifiable modifiable cause | NA | NA | NA |
| Silverman, 2003[^105^](#_ENREF_105) | Consensus statement | To discuss a multifaceted approach to reducing preventable adverse drug events | NA | NA | Author-defined | Adverse drug events | Presence of an identifiable modifiable cause | NA | NA | NA |
| Simpson, 2011[^106^](#_ENREF_106) | Opinion piece | A review of current issues in perinatal patient safety and quality | Moms and neonates; perinatal period | Inpatient | Consensus | Various | Reasonable adaptation to a process will prevent future recurrence | NA | NA | NA |
| Stone, 2009[^107^](#_ENREF_107) | Opinion piece | To describe a joint initative between the NHS and the pharmaceutical industry that aims to address preventable harm from medicine allergy | Patients with medication allergy | All settings | Author-defined | Adverse drug events | Presence of an identifiable modifiable cause | NA | NA | NA |
| Thomas, 2009[^108^](#_ENREF_108) | R, Obs | To examine the magnitude of potentially avoidable iatrogenic complications of male urethral catheterization (UC) within a tertiary-care supra-regional teaching hospital | Males who underwent urinary catheterization | Inpatient | Author-defined | (Other) Urethral trauma | Reasonable adaptation to a process will prevent future recurrence | NA | NA | NA |
| Thomsen, 2007[^109^](#_ENREF_109) | SR | To estimate the incidence and describe characteristics of preventable drug events in ambulatory care | Outpatients | Outpatient | Consensus | Adverse drug events | Presence of an identifiable modifiable cause | NA | NA | NA |
| Thornlow, 2006[^110^](#_ENREF_110) | Cross | To examined the statistical relationship between hospital ownership and teaching status and hospital rates for potentially preventable adverse events measured using patient safety indicators | Hospitalized patients | Inpatient | Consensus | Various | Morbidity adjusted risk estimate | NA | NA | NA |
| Undre, 2009[^111^](#_ENREF_111) | Opinion piece | To review measures that assess technical as well as nontechnical skill in surgical teams and examine their existing applications and potential applicability to urological surgery | Patients undergoing urologic surgery | Inpatient | Consensus | Various | Presence of an identifiable modifiable cause | NA | NA | NA |
| Van Doormaal, 2009[^112^](#_ENREF_112) | Cross | To compare Computerised Physician Order Entry (CPOE) and Clinical Decision Support Systems (CDSS) | Inpatients | Inpatient | Author-defined | Adverse drug events | Presence of an identifiable modifiable cause | NA | NA | NA |
| Van Doormaal, 2009, JAMIA[^113^](#_ENREF_113) | P, Obs | To evaluate the effect of a Computerized Physician Order Entry system with basic Clinical Decision Support (CPOE/CDSS) on the incidence of medication errors (MEs) and preventable adverse drug events (pADEs) | Inpatients | Inpatient | Author-defined | Adverse drug events | Presence of an identifiable modifiable cause | NA | NA | NA |
| Van Doormal, 2009[^114^](#_ENREF_114) | P, Obs | To determine the impact of the various types of prescribing and transcribing errors on preventable adverse drug events in hospitalized patients | Hospitalized patients | Inpatient | Consensus | Adverse drug events | Presence of an identifiable modifiable cause | NA | NA | NA |
| Van Doormal, 2008[^115^](#_ENREF_115) | P, Obs | To determine the reliability of the assessment of preventable adverse drug events in daily practice | Hospitalized patients | Inpatient | Consensus | Adverse drug events | Presence of an identifiable modifiable cause | Data were collected from 5 internal medicine wards (1900 beds in total) | Kappa calculation ranged from 0.36 for physicians to 0.49 for pharmacists | NA |
| Vanderheyden, 2005[^116^](#_ENREF_116) | Cross | To analyze the results of the Alberta Patient Safety Survey 2004 | Albertans | NA | Author-defined | Various | Presence of an identifiable modifiable cause | 1500 adult Albertans were surveyed | 81% inter-rater agreement | Preventable errors were defined as resulting in serious harm, such as death, disability or additional prolonged treatment that occurred while receiving medical care |
| Vogel, 2009[^117^](#_ENREF_117) | Cross | To determine recent trends in sepsis incidence, severity, and mortality rate after surgical procedures and to evaluate changes in the pattern of septicemia pathogens over time | Post-op hospitalized patients | Inpatient | Author-defined | (Other) Postoperative sepsis, death | Presence of an identifiable modifiable cause | NA | NA | NA |
| Von Laue, 2003[^118^](#_ENREF_118) | SR | To review the literature of adverse drug events and their preventability and to report on their incidence, characteristics, risk factors, costs and preventive strategies | Hospitalized patients | Inpatient | Peer-reviewed citation | Adverse drug events | Presence of an identifiable modifiable cause | NA | NA | NA |
| Wachter, 2009[^119^](#_ENREF_119) | Opinion piece | A commentary on patient safety | NA | NA | Peer-reviewed citation | Various | Adherence to guidelines | NA | NA | NA |
| Warwick, 2007[^120^](#_ENREF_120) | R, Obs | to determine the time courses of both the incidence of venous thromboembolism and effective prophylaxis | Hospitalized patients | Inpatient | Peer-reviewed citation | Hospital-stay related venous thromboembolism | Adherence to guidelines | NA | NA | NA |
| Winters, 2011[^121^](#_ENREF_121) | Opinion piece | A commentary on diagnostic errors and the role of checklists | NA | NA | Author-defined | Diagnostic error | Reasonable adaptation to a process will prevent future recurrence | NA | NA | NA |
| Winterstein, 2002[^122^](#_ENREF_122) | Cross | To analyze the ability of a hospital's adverse drug reaction database to identify common and repeated patterns of preventable adverse drug events | Inpatients | Inpatient | Author-defined | Adverse drug events | Presence of an identifiable modifiable cause | NA | NA | NA |
| Woods, 2005[^123^](#_ENREF_123) | R, Obs | To describe the incidence and types of adverse events and preventable adverse events in children | Hospitalized children | Inpatient | Author-defined | Various | Presence of an identifiable modifiable cause | Over 11,000 hospital discharged patients from all the hospitals in Utah and Colorado were analyzed | Agreement was 79% (Kappa=0.4) | NA |
| Woods, 2007[^124^](#_ENREF_124) | Cross | To describe the epidemiology of errors and adverse events in ambulatory care |  | Outpatient | Peer-reviewed citation | Various | Presence of an identifiable modifiable cause | 14,700 hospital discharge records were reviewed and 587 adverse events were identified | Kappa=0.8 | NA |
| Zafarghandi, 2003[^125^](#_ENREF_125) | P, Obs | To estimate the number of preventable trauma deaths in teaching hospitals in Tehran | Patients who died secondary to trauma | Inpatient | Peer-reviewed citation | Death | Presence of an identifiable modifiable cause | NA | NA | NA |
| Zandieh, 2008[^126^](#_ENREF_126) | P, Obs | To determine whether there are racial/ethnic, socioeconomic, parental linguistic, or parental educational disparities in children who experienced an adverse drug event (ADE) in the ambulatory setting | Children < 21 years | Outpatient | Peer-reviewed citation | Adverse drug events | Reasonable adaptation to a process will prevent future recurrence | 1689 patient prescription were analyzed | Kappa=0.95 | Variable severity included. None of the preventable ADEs was life threatening or fatal,  14% were serious, and 86% were significant. |
| Zegers, 2009[^127^](#_ENREF_127) | R, Obs | To determine the incidence, type, nature and preventability of adverse events in hospitalized patients in the Netherlands | Hospitalized patients | Inpatient | Author-defined | Various | Presence of an identifiable modifiable cause | 7926 admissions were reviewed | Agreement was 70% with a kappa=0.40 | NA |

^a^Cross: cross-sectional; ^b^R Obs: Observational retrospective; ^c^P Obs: Observational prospective

**1.** Arzy S, Brezis M, Khoury S, Simon SR, Ben-Hur T. Misleading one detail: a preventable mode of diagnostic error? *J Eval Clin Pract.* Oct 2009;15(5):804-806.

**2.** Aspden. IOM report: Patient safety - Achieving a new standard for care. *Acad Emerg Med.* Oct 2005;12(10):1011-1012.

**3.** Baker GR, Norton PG, Flintoft V, et al. The Canadian Adverse Events Study: the incidence of adverse events among hospital patients in Canada. *Cmaj.* May 25 2004;170(11):1678-1686.

**4.** Bapoje SR, Gaudiani JL, Narayanan V, Albert RK. Unplanned transfers to a medical intensive care unit: causes and relationship to preventable errors in care. *J Hosp Med.* Feb 2011;6(2):68-72.

**5.** Bartlett G, Blais R, Tamblyn R, Clermont RJ, MacGibbon B. Impact of patient communication problems on the risk of preventable adverse events in acute care settings. *Cmaj.* Jun 3 2008;178(12):1555-1562.

**6.** Beckmann U, Bohringer C, Carless R, et al. Evaluation of two methods for quality improvement in intensive care: facilitated incident monitoring and retrospective medical chart review. *Crit Care Med.* Apr 2003;31(4):1006-1011.

**7.** Berenholtz SM, Pronovost PJ. Monitoring patient safety. *Crit Care Clin.* Jul 2007;23(3):659-673.

**8.** Berenholtz SM, Pham JC, Thompson DA, et al. Collaborative cohort study of an intervention to reduce ventilator-associated pneumonia in the intensive care unit. *Infect Control Hosp Epidemiol.* Apr 2011;32(4):305-314.

**9.** Brilli RJ, McClead RE, Jr., Davis T, Stoverock L, Rayburn A, Berry JC. The Preventable Harm Index: an effective motivator to facilitate the drive to zero. *J Pediatr.* Oct 2010;157(4):681-683.

**10.** Brooke BS, Meguid RA, Makary MA, Perler BA, Pronovost PJ, Pawlik TM. Improving surgical outcomes through adoption of evidence-based process measures: intervention specific or associated with overall hospital quality? *Surgery.* Apr 2010;147(4):481-490.

**11.** Buckley MS, Erstad BL, Kopp BJ, Theodorou AA, Priestley G. Direct observation approach for detecting medication errors and adverse drug events in a pediatric intensive care unit. *Pediatr Crit Care Med.* Mar 2007;8(2):145-152.

**12.** Burda SA, Hobson D, Pronovost PJ. What is the patient really taking? Discrepancies between surgery and anesthesiology preoperative medication histories. *Qual Saf Health Care.* Dec 2005;14(6):414-416.

**13.** Canale ST. Wrong-site surgery: a preventable complication. *Clin Orthop.* Apr 2005(433):26-29.

**14.** Carpenter KB, Duevel MA, Lee PW, et al. Measures of patient safety in developing and emerging countries: a review of the literature. *Qual Saf Health Care.* Feb 2010;19(1):48-54.

**15.** Catalano K. Preventable hospital-acquired conditions: the whys and wherefores. *Plast Surg Nurs.* Jul-Sep 2008;28(3):158-161.

**16.** Chang DC, Handly N, Abdullah F, et al. The occurrence of potential patient safety events among trauma patients: are they random? *Ann Surg.* Feb 2008;247(2):327-334.

**17.** Chuang Y-T, Ginsburg L, Berta WB. Learning from preventable adverse events in health care organizations: development of a multilevel model of learning and propositions. *Health Care Manage Rev.* Oct-Dec 2007;32(4):330-340.

**18.** Cohen AT, Tapson VF, Bergmann JF, et al. Venous thromboembolism risk and prophylaxis in the acute hospital care setting (ENDORSE study): a multinational cross-sectional study. *Lancet.* Feb 2 2008;371(9610):387-394.

**19.** Cooper JB, Newbower RS, Long CD, McPeek B. Preventable anesthesia mishaps: a study of human factors. 1978. *Qual Saf Health Care.* Sep 2002;11(3):277-282.

**20.** Cupryk M. Standardizing patient safety risk management. *Pharmaceutical Engineering.* March-April 2011;31(2):66-76.

**21.** Dagi TF, Berguer R, Moore S, Reines HD. Preventable errors in the operating room--part 2: retained foreign objects, sharps injuries, and wrong site surgery. *Curr Probl Surg.* Jun 2007;44(6):352-381.

**22.** Daniels JP, King AD, Cochrane DD, et al. A human factors and survey methodology-based design of a web-based adverse event reporting system for families. *Int J Med Inf.* May 2010;79(5):339-348.

**23.** Davis P, Lay-Yee R, Briant R, Scott A. Preventable in-hospital medical injury under the "no fault" system in New Zealand. *Qual Saf Health Care.* Aug 2003;12(4):251-256.

**24.** De Wet C, Bowie P. Screening electronic patient records to detect preventable harm: a trigger tool for primary care. *Qual Prim Care.* 2011;19(2):115-125.

**25.** DePalo VA, McNicoll L, Cornell M, Rocha JM, Adams L, Pronovost PJ. The Rhode Island ICU collaborative: a model for reducing central line-associated bloodstream infection and ventilator-associated pneumonia statewide. *Qual Saf Health Care.* Dec 2010;19(6):555-561.

**26.** Devine EB, Hansen RN, Wilson-Norton JL, et al. The impact of computerized provider order entry on medication errors in a multispecialty group practice. *J Am Med Inform Assoc.* Jan-Feb 2010;17(1):78-84.

**27.** Dupuis O, Dupont C, Gaucherand P, et al. Is neonatal neurological damage in the delivery room avoidable? Experience of 33 levels I and II maternity units of a French perinatal network. *Eur J Obstet Gynecol Reprod Biol.* Sep 2007;134(1):29-36.

**28.** Elder NC, Dovey SM. Classification of medical errors and preventable adverse events in primary care: a synthesis of the literature. *J.* Nov 2002;51(11):927-932.

**29.** Friedley NJ. Rx for medication errors. A patient medication safety plan can help prevent the cascade of devastating and preventable complications from adverse drug events. *Med Econ.* Oct 17 2008;85(20):34-38.

**30.** Friedman RJ, Gallus AS, Cushner FD, Fitzgerald G, Anderson FA, Jr. Physician compliance with guidelines for deep-vein thrombosis prevention in total hip and knee arthroplasty. *Curr Med Res Opin.* Jan 2008;24(1):87-97.

**31.** Garner JD. Adverse drug events in nursing homes: common and preventable. *J Women Aging.* 2001;13(3):1-3.

**32.** Gibbs VC, Coakley FD, Reines HD. Preventable errors in the operating room: retained foreign bodies after surgery--Part I. *Curr Probl Surg.* May 2007;44(5):281-337.

**33.** Gilbert GL, Cheung PY, Kerridge IB. Infection control, ethics and accountability. *Med J Aust.* Jun 15 2009;190(12):696-698.

**34.** Greene S, Dargan P, Shin GY, Jones AI. Doctors and nurses estimation of the weight of patients: A preventable source of systematic error. *J Toxicol Clin Toxicol.* 2004;42(5):611-615.

**35.** Gruen RL, Jurkovich GJ, McIntyre LK, Foy HM, Maier RV. Patterns of errors contributing to trauma mortality: lessons learned from 2,594 deaths. *Ann Surg.* Sep 2006;244(3):371-380.

**36.** Gurses AP, Marsteller JA, Ozok AA, Xiao Y, Owens S, Pronovost PJ. Using an interdisciplinary approach to identify factors that affect clinicians' compliance with evidence-based guidelines. *Crit Care Med.* Aug 2010;38(8 Suppl):S282-291.

**37.** Halfon P, Eggli Y, Matter M, Kallay C, van Melle G, Burnand B. Risk-adjusted rates for potentially avoidable reoperations were computed from routine hospital data. *J Clin Epidemiol.* Jan 2007;60(1):56-67.

**38.** Haller G, Myles PS, Langley M, Stoelwinder J, McNeil J. Assessment of an unplanned admission to the intensive care unit as a global safety indicator in surgical patients. *Anaesthesia and Intensive Care.* Mar 2008;36(2):190-200.

**39.** Heitmiller E, Martinez E, Pronovost PJ. Identifying and learning from mistakes. *Anesthesiology.* Apr 2007;106(4):654-656.

**40.** Hofer TP, Hayward RA. Are bad outcomes from questionable clinical decisions preventable medical errors? A case of cascade iatrogenesis. *Ann Intern Med.* Sep 3 2002;137(5 Part 1):327-333.

**41.** Hoff TJ, Soerensen C. No payment for preventable complications: reviewing the early literature for content, guidance, and impressions. *Qual Manag Health Care.* Jan-Mar 2011;20(1):62-75.

**42.** Holzmueller CG, Pronovost PJ, Dickman F, et al. Creating the web-based intensive care unit safety reporting system. *J Am Med Inform Assoc.* Mar-Apr 2005;12(2):130-139.

**43.** Hoonhout LHF, de Bruijne MC, Wagner C, Asscheman H, van der Wal G, van Tulder MW. Nature, occurrence and consequences of medication-related adverse events during hospitalization: a retrospective chart review in the Netherlands. *Drug Saf.* Oct 1 2010;33(10):853-864.

**44.** Howard RL, Avery AJ, Howard PD, Partridge M. Investigation into the reasons for preventable drug related admissions to a medical admissions unit: observational study. *Qual Saf Health Care.* Aug 2003;12(4):280-285.

**45.** Hug BL, Witkowski DJ, Sox CM, et al. Occurrence of adverse, often preventable, events in community hospitals involving nephrotoxic drugs or those excreted by the kidney. *Kidney Int.* Dec 2009;76(11):1192-1198.

**46.** Hughes JS, Averill RF, Goldfield NI, et al. Identifying potentially preventable complications using a present on admission indicator. *Health Care Financ Rev.* 2006;27(3):63-82.

**47.** Ibrahim JE, Ranson DL, O'Brien A, Charles A, Young C. Forensic investigation of medical treatment related deaths. *Leg Med (Tokyo).* Apr 2009;11 Suppl 1:S71-75.

**48.** Iyengar A, Baxter A, Forster AJ. Using Medical Emergency Teams to detect preventable adverse events. *Crit Care.* 2009;13(4):R126.

**49.** Jennings HR, Miller EC, Williams TS, Tichenor SS, Woods EA. Reducing anticoagulant medication adverse vents and avoidable patient harm. *Jt Comm J Qual Patient Saf.* Apr 2008;34(4):196-200.

**50.** Kaissi A, Johnson T, Kirschbaum MS. Measuring teamwork and patient safety attitudes of high-risk areas. *Nurs Econ.* Sep-Oct 2003;21(5):211-218.

**51.** Kanjanarat P, Winterstein AG, Johns TE, Hatton RC, Gonzalez-Rothi R, Segal R. Nature of preventable adverse drug events in hospitals: a literature review. *Am J Health-Syst Pharm.* Sep 1 2003;60(17):1750-1759.

**52.** Karsh B-T, Brown R. Macroergonomics and patient safety: the impact of levels on theory, measurement, analysis and intervention in patient safety research. *Appl Ergon.* Sep 2010;41(5):674-681.

**53.** Kleinpell R. Evidence-based review and discussion points. Certification and patient safety. *Am J Crit Care.* Mar 2009;18(2):115-116.

**54.** Kucukarslan SN, Peters M, Mlynarek M, Nafziger DA. Pharmacists on rounding teams reduce preventable adverse drug events in hospital general medicine units. *Arch Intern Med.* Sep 22 2003;163(17):2014-2018.

**55.** Kunac DL, Reith DM. Preventable medication-related events in hospitalised children in New Zealand. *N Z Med J.* Apr 18 2008;121(1272):17-32.

**56.** Kuzel AJ, Woolf SH, Gilchrist VJ, et al. Patient reports of preventable problems and harms in primary health care. *Ann Fam Med.* Jul-Aug 2004;2(4):333-340.

**57.** Laditka SB, Laditka JN, Probst JC. Racial and ethnic disparities in potentially avoidable delivery complications among pregnant Medicaid beneficiaries in South Carolina. *Matern Child Health J.* Jul 2006;10(4):339-350.

**58.** Larsen GY, Donaldson AE, Parker HB, Grant MJC. Preventable harm occurring to critically ill children. *Pediatr Crit Care Med.* Jul 2007;8(4):331-336.

**59.** Lee BH, Lehmann CU, Jackson EV, et al. Assessing controlled substance prescribing errors in a pediatric teaching hospital: an analysis of the safety of analgesic prescription practice in the transition from the hospital to home. *J Pain.* Feb 2009;10(2):160-166.

**60.** Lessing C, Schmitz A, Albers B, Schrappe M. Impact of sample size on variation of adverse events and preventable adverse events: systematic review on epidemiology and contributing factors. *Qual Saf Health Care.* Dec 2010;19(6):e24.

**61.** Lilly CM, Cody S, Zhao H, et al. Hospital mortality, length of stay, and preventable complications among critically ill patients before and after tele-ICU reengineering of critical care processes. *Jama.* Jun 1 2011;305(21):2175-2183.

**62.** Lu TC, Tsai CL, Lee CC, et al. Preventable deaths in patients admitted from emergency department. *Emerg Med J.* Jun 2006;23(6):452-455.

**63.** Mackinnon NJ, Hepler CD. Indicators of preventable drug-related morbidity in older adults 2. Use within a managed care organization. *J Manage Care Pharm.* Mar-Apr 2003;9(2):134-141.

**64.** Makary MA, Mukherjee A, Sexton JB, et al. Operating room briefings and wrong-site surgery. *J Am Coll Surg.* Feb 2007;204(2):236-243.

**65.** McBride SC, Chiang VW, Goldmann DA, Landrigan CP. Preventable adverse events in infants hospitalized with bronchiolitis. *Pediatrics.* Sep 2005;116(3):603-608.

**66.** McCulloch P. Safe surgery-how can we improve our performance in the operating theatre? *European Journal of Surgical Oncology.* 2010;36 (9):843.

**67.** Michaels RK, Makary MA, Dahab Y, et al. Achieving the National Quality Forum's "Never Events": prevention of wrong site, wrong procedure, and wrong patient operations. *Ann Surg.* Apr 2007;245(4):526-532.

**68.** Michel P, Quenon JL, de Sarasqueta AM, Scemama O. Comparison of three methods for estimating rates of adverse events and rates of preventable adverse events in acute care hospitals. *Bmj.* Jan 24 2004;328(7433):199.

**69.** Miller MR, Robinson KA, Lubomski LH, Rinke ML, Pronovost PJ. Medication errors in paediatric care: a systematic review of epidemiology and an evaluation of evidence supporting reduction strategy recommendations. *Qual Saf Health Care.* Apr 2007;16(2):116-126.

**70.** Millman EA, Pronovost PJ, Makary MA, Wu AW. Patient-assisted incident reporting: including the patient in patient safety. *J Patient Saf.* Jun 2011;7(2):106-108.

**71.** Morriss FH, Jr., Abramowitz PW, Nelson SP, et al. Effectiveness of a barcode medication administration system in reducing preventable adverse drug events in a neonatal intensive care unit: a prospective cohort study. *J Pediatr.* Mar 2009;154(3):363-368, 368.e361.

**72.** Murphy DJ, Needham DM, Goeschel C, Fan E, Cosgrove SE, Pronovost PJ. Monitoring and reducing central line-associated bloodstream infections: a national survey of state hospital associations. *Am J Med Qual.* Jul-Aug 2010;25(4):255-260.

**73.** Murphy DJ, Pronovost PJ. Reducing preventable harm: comment on "Clinical and economic outcomes attributable to health care-associated sepsis and pneumonia". *Arch Intern Med.* Feb 22 2010;170(4):353-355.

**74.** Naessens JM, Campbell CR, Huddleston JM, et al. A comparison of hospital adverse events identified by three widely used detection methods. *Int J Qual Health Care.* Aug 2009;21(4):301-307.

**75.** Needham DM, Thompson DA, Holzmueller CG, et al. A system factors analysis of airway events from the Intensive Care Unit Safety Reporting System (ICUSRS). *Crit Care Med.* Nov 2004;32(11):2227-2233.

**76.** Needham DM, Sinopoli DJ, Thompson DA, et al. A system factors analysis of "line, tube, and drain" incidents in the intensive care unit. *Crit Care Med.* Aug 2005;33(8):1701-1707.

**77.** Newman-Toker DE, Pronovost PJ. Diagnostic errors--the next frontier for patient safety. *Jama.* Mar 11 2009;301(10):1060-1062.

**78.** Newton RC, Mytton OT, Aggarwal R, et al. Making existing technology safer in healthcare. *Qual Saf Health Care.* Aug 2010;19 Suppl 2:i15-24.

**79.** O'Grady NP, Gerberding JL, Weinstein RA, Masur H. Patient safety and the science of prevention: the time for implementing the Guidelines for the prevention of intravascular catheter-related infections is now. *Crit Care Med.* Jan 2003;31(1):291-292.

**80.** Pandey P, Chaudhary R, Tondon R, Khetan D. Predictable and avoidable human errors in phlebotomy area - an exclusive analysis from a tertiary health care system blood bank. *Transfus Med.* Oct 2007;17(5):375-378.

**81.** Peterson JF, Bates DW. Preventable medication errors: identifying and eliminating serious drug interactions. *J Am Pharm Assoc (Wash).* Mar-Apr 2001;41(2):159-160.

**82.** Pham JC, Story JL, Hicks RW, et al. National study on the frequency, types, causes, and consequences of voluntarily reported Emergency Department medication errors. *Journal of Emergency Medicine.* May 2011;40(5):485-492.

**83.** SSI BCG vaccine: avoidable adverse effects. *Prescrire Int.* Oct 2007;16(91):200-201.

**84.** Oral methotrexate: preventing avoidable overdose. *Prescrire Int.* Aug 2007;16(90):150-152.

**85.** Pronovost PJ, Wu AW, Sexton JB. Acute decompensation after removing a central line: practical approaches to increasing safety in the intensive care unit. *Ann Intern Med.* Jun 15 2004;140(12):1025-1033.

**86.** Pronovost PJ, Thompson DA, Holzmueller CG, Lubomski LH, Morlock LL. Defining and measuring patient safety. *Crit Care Clin.* Jan 2005;21(1):1-19, vii.

**87.** Pronovost PJ, Thompson DA, Holzmueller CG, et al. Toward learning from patient safety reporting systems. *J Crit Care.* Dec 2006;21(4):305-315.

**88.** Pronovost PJ, Colantuoni E. Measuring preventable harm: helping science keep pace with policy. *Jama.* Mar 25 2009;301(12):1273-1275.

**89.** Pronovost PJ. We need leaders: The 48th Annual Rovenstine Lecture. *Anesthesiology.* Apr 2010;112(4):779-785.

**90.** Pronovost PJ, Goeschel CA, Colantuoni E, et al. Sustaining reductions in catheter related bloodstream infections in Michigan intensive care units: observational study. *Bmj.* 2010;340:c309.

**91.** Pronovost PJ, Cardo DM, Goeschel CA, Berenholtz SM, Saint S, Jernigan JA. A research framework for reducing preventable patient harm. *Clin Infect Dis.* Feb 2011;52(4):507-513.

**92.** Rabinowitz E. Preventing the preventable. Health plans implement high-tech and low-tech solutions aimed at reducing hospital medication errors. *AHIP Cover.* Nov-Dec 2008;49(6):26-32.

**93.** Rahme E, Dasgupta K, Burman M, et al. Postdischarge thromboprophylaxis and mortality risk after hip-or knee-replacement surgery. *Cmaj.* Jun 3 2008;178(12):1545-1554.

**94.** Robbins J. Hospital checklists. Transforming evidence-based care and patient safety protocols into routine practice. *Crit Care Nurs Q.* Apr-Jun 2011;34(2):142-149.

**95.** ICU patients at risk for preventable errors. *Hosp Case Manag.* Dec 2005;13(12):184-185.

**96.** Sadri A, Braithwaite IJ, Abdul-Jabar HB, Sarraf KM. Understanding of intra-operative tourniquets amongst orthopaedic surgeons and theatre staff--a questionnaire study. *Ann R Coll Surg Engl.* Apr 2010;92(3):243-245; quiz 241p following 245.

**97.** Sawyer M, Weeks K, Goeschel CA, et al. Using evidence, rigorous measurement, and collaboration to eliminate central catheter-associated bloodstream infections. *Crit Care Med.* August 2010;38(8 SUPPL.):S292-S298.

**98.** Schlack WS, Boermeester MA. Patient safety during anaesthesia: incorporation of the WHO safe surgery guidelines into clinical practice. *Curr Opin Anaesthesiol.* Dec 2010;23(6):754-758.

**99.** Segal AJ, Bush WH, Jr. Avoidable errors in dealing with anaphylactoid reactions to iodinated contrast media. *Invest Radiol.* Mar 2011;46(3):147-151.

**100.** Seiden SC, Barach P. Wrong-side/wrong-site, wrong-procedure, and wrong-patient adverse events: Are they preventable? *Arch Surg.* Sep 2006;141(9):931-939.

**101.** Selby R, Bijan J. Borah P, Heather P. McDonald M, Henry J. Henk P, Mark Crowther M, Philip S. Wells M. Impact of Thromboprophylaxis Guidelines on Clinical Outcomes Following Total Hip and Total Knee Replacement. 2011.

**102.** Seliger SL, Zhan M, Hsu VD, Walker LD, Fink JC. Chronic kidney disease adversely influences patient safety. *J Am Soc Nephrol.* Dec 2008;19(12):2414-2419.

**103.** Sexton JB, Berenholtz SM, Goeschel CA, et al. Assessing and improving safety climate in a large cohort of intensive care units. *Crit Care Med.* May 2011;39(5):934-939.

**104.** Sherman FT. Treating your mother? A new type of preventable medical error. *Geriatrics.* Jul 2008;63(7):10-11.

**105.** Silverman JB, Stapinski CD, Churchill WW, Neppl C, Bates DW, Gandhi TK. Multifaceted approach to reducing preventable adverse drug events. *Am J Health-Syst Pharm.* Mar 15 2003;60(6):582-586.

**106.** Simpson KR. Perinatal patient safety and quality. *J Perinat Neonatal Nurs.* Apr-Jun 2011;25(2):103-107.

**107.** Stone M, Tomlin S, Wilcock M. How to reduce preventable harm from medicine allergy - A pilot study. *Pharmaceutical Journal.* 20 Jun 2009;282(7557):745-746.

**108.** Thomas AZ, Giri SK, Meagher D, Creagh T. Avoidable iatrogenic complications of urethral catheterization and inadequate intern training in a tertiary-care teaching hospital. *BJU Int.* Oct 2009;104(8):1109-1112.

**109.** Thomsen LA, Winterstein AG, Sondergaard B, Haugbolle LS, Melander A. Systematic review of the incidence and characteristics of preventable adverse drug events in ambulatory care. *Ann Pharmacother.* Sep 2007;41(9):1411-1426.

**110.** Thornlow DK, Stukenborg GJ. The association between hospital characteristics and rates of preventable complications and adverse events. *Med Care.* Mar 2006;44(3):265-269.

**111.** Undre S, Arora S, Sevdalis N. Surgical performance, human error and patient safety in urological surgery. *British Journal of Medical and Surgical Urology.* January 2009;2(1):2-10.

**112.** Van Doormaal J, Rommers M, Kosterink J, Teepe-Twiss I, Haaijer-Ruskamp F, Mol P. Comparing methods to identify patients at risk of medication related harm. *Pharmacoepidemiology and Drug Safety (PDS).* 2009;18 (S1):S243.

**113.** van Doormaal JE, van den Bemt PMLA, Zaal RJ, et al. The influence that electronic prescribing has on medication errors and preventable adverse drug events: an interrupted time-series study. *J Am Med Inform Assoc.* Nov-Dec 2009;16(6):816-825.

**114.** van Doormaal JE, van den Bemt PMLA, Mol PGM, et al. Medication errors: the impact of prescribing and transcribing errors on preventable harm in hospitalised patients. *Qual Saf Health Care.* Feb 2009;18(1):22-27.

**115.** van Doormaal JE, Mol PGM, van den Bemt PMLA, et al. Reliability of the assessment of preventable adverse drug events in daily clinical practice. *Pharmacoepidemiol Drug Saf.* Jul 2008;17(7):645-654.

**116.** Vanderheyden LC, Northcott HC, Adair CE, et al. Reports of preventable medical errors from the Alberta Patient Safety Survey 2004. *Healthc Q.* 2005;8 Spec No:107-114.

**117.** Vogel TR, Dombrovskiy VY, Lowry SF. Trends in postoperative sepsis: are we improving outcomes? *Surg Infect (Larchmt).* Feb 2009;10(1):71-78.

**118.** von Laue NC, Schwappach DLB, Koeck CM. The epidemiology of preventable adverse drug events: a review of the literature. *Wien Klin Wochenschr.* Jul 15 2003;115(12):407-415.

**119.** Wachter RM, Pronovost PJ. Balancing "no blame" with accountability in patient safety. *N Engl J Med.* 01 Oct 2009;361(14):1401-1406+1328.

**120.** Warwick D, Friedman RJ, Agnelli G, et al. Insufficient duration of venous thromboembolism prophylaxis after total hip or knee replacement when compared with the time course of thromboembolic events: findings from the Global Orthopaedic Registry. *J Bone Joint Surg Br.* Jun 2007;89(6):799-807.

**121.** Winters BD, Aswani MS, Pronovost PJ. Commentary: Reducing diagnostic errors: another role for checklists? *Acad Med.* Mar 2011;86(3):279-281.

**122.** Winterstein AG, Hatton RC, Gonzalez-Rothi R, Johns TE, Segal R. Identifying clinically significant preventable adverse drug events through a hospital's database of adverse drug reaction reports. *Am J Health-Syst Pharm.* Sep 15 2002;59(18):1742-1749.

**123.** Woods D, Thomas E, Holl J, Altman S, Brennan T. Adverse events and preventable adverse events in children. *Pediatrics.* Jan 2005;115(1):155-160.

**124.** Woods DM, Thomas EJ, Holl JL, Weiss KB, Brennan TA. Ambulatory care adverse events and preventable adverse events leading to a hospital admission. *Qual Saf Health Care.* Apr 2007;16(2):127-131.

**125.** Zafarghandi M-R, Modaghegh M-HS, Roudsari BS. Preventable trauma death in Tehran: an estimate of trauma care quality in teaching hospitals. *J Trauma.* Sep 2003;55(3):459-465.

**126.** Zandieh SO, Goldmann DA, Keohane CA, Yoon C, Bates DW, Kaushal R. Risk factors in preventable adverse drug events in pediatric outpatients. *J Pediatr.* Feb 2008;152(2):225-231.

**127.** Zegers M, de Bruijne MC, Wagner C, et al. Adverse events and potentially preventable deaths in Dutch hospitals: results of a retrospective patient record review study. *Qual Saf Health Care.* Aug 2009;18(4):297-302.
