# Supplementary material for: What is preventable harm in healthcare? A systematic review of definitions
Source: BMC Health Serv Res. 2012 May 25;12:128. doi: 10.1186/1472-6963-12-128 (PMC3405467; doi:10.1186/1472-6963-12-128)
Supplement: Additional file 3 — Table S2. Number of articles reported under each definition, broken up by the type of harm. [file 1472-6963-12-128-S3.docx]

Supplemental Table 2: Number of articles reported under each definition, broken up by the type of harm.

|  | Various | Adverse drug events | Other | CLABSI^*^ | Hospital-stay related venous thromboembolism | Health care-associated infections | Wrong-site surgery | Death | Diagnostic error | Total |
| --- | --- | --- | --- | --- | --- | --- | --- | --- | --- | --- |
| Historical comparison | 1 | 1 | n/a | n/a | n/a | n/a | n/a | n/a | n/a | 2 |
| Comparison with another cohort | 2 | n/a | n/a | 1 | n/a | n/a | n/a | n/a | n/a | 3 |
| Morbidity adjusted risk estimate | 4 | 1 | 3 | n/a | n/a | n/a | n/a | 1 | n/a | 9 |
| Adherence to guidelines | 6 | 2 | 3 | 2 | 4 | 3 | 1 | 1 | n/a | 22 |
| Reasonable adaptation to a process will prevent future recurrence | 10 | 8 | 7 | 2 | 1 | 1 | n/a | n/a | 1 | 30 |
| The Presence of an identifiable modifiable cause | 18 | 20 | 13 | 2 | n/a | n/a | 1 | 2 | 2 | 58 |
| All harm is preventable | 1 | 1 | 4 | n/a | n/a | n/a | 2 | n/a | n/a | 8 |
| Total | 42 | 33 | 30 | 7 | 5 | 4 | 4 | 4 | 3 | 132 |

^*^CLABSI: central-line associated bloodstream infections
